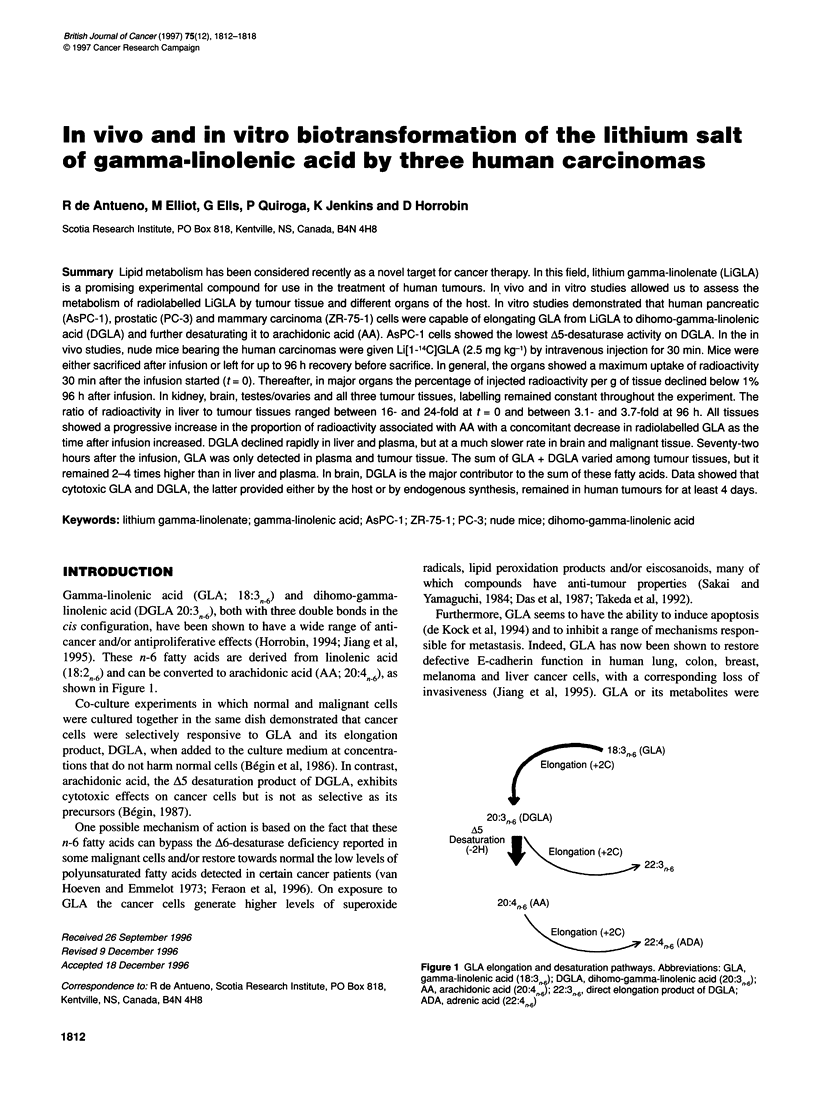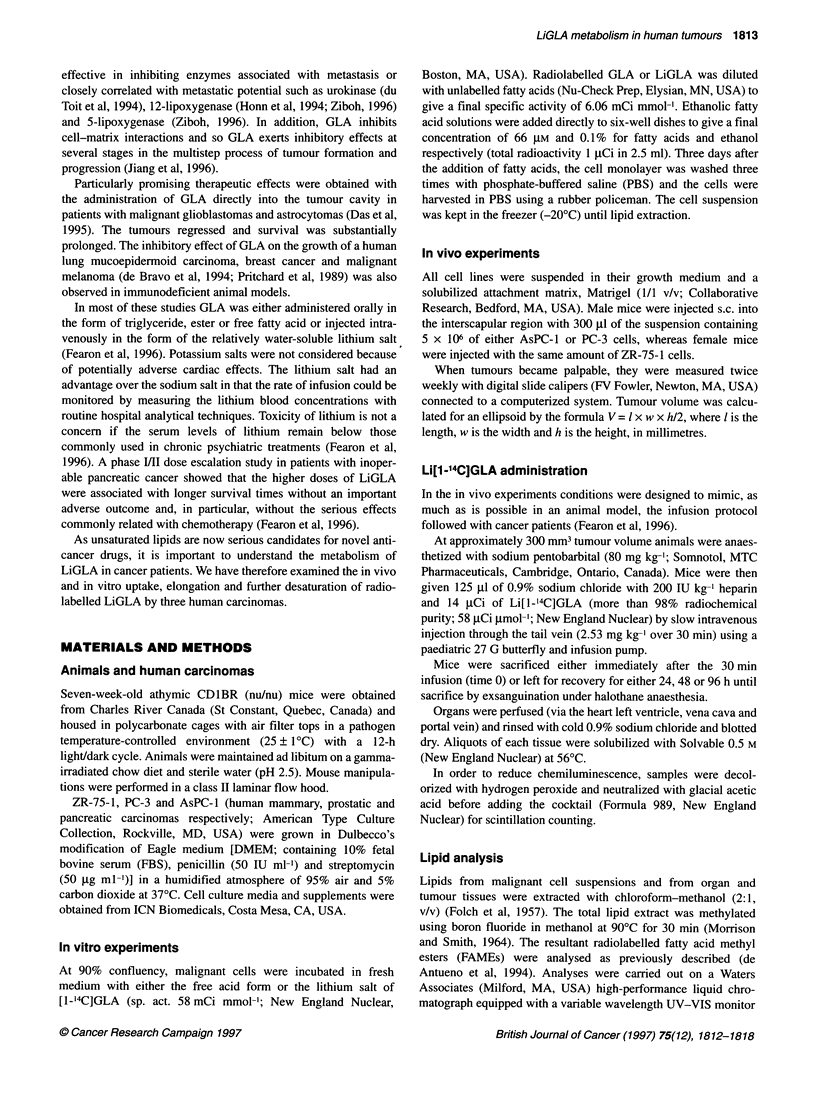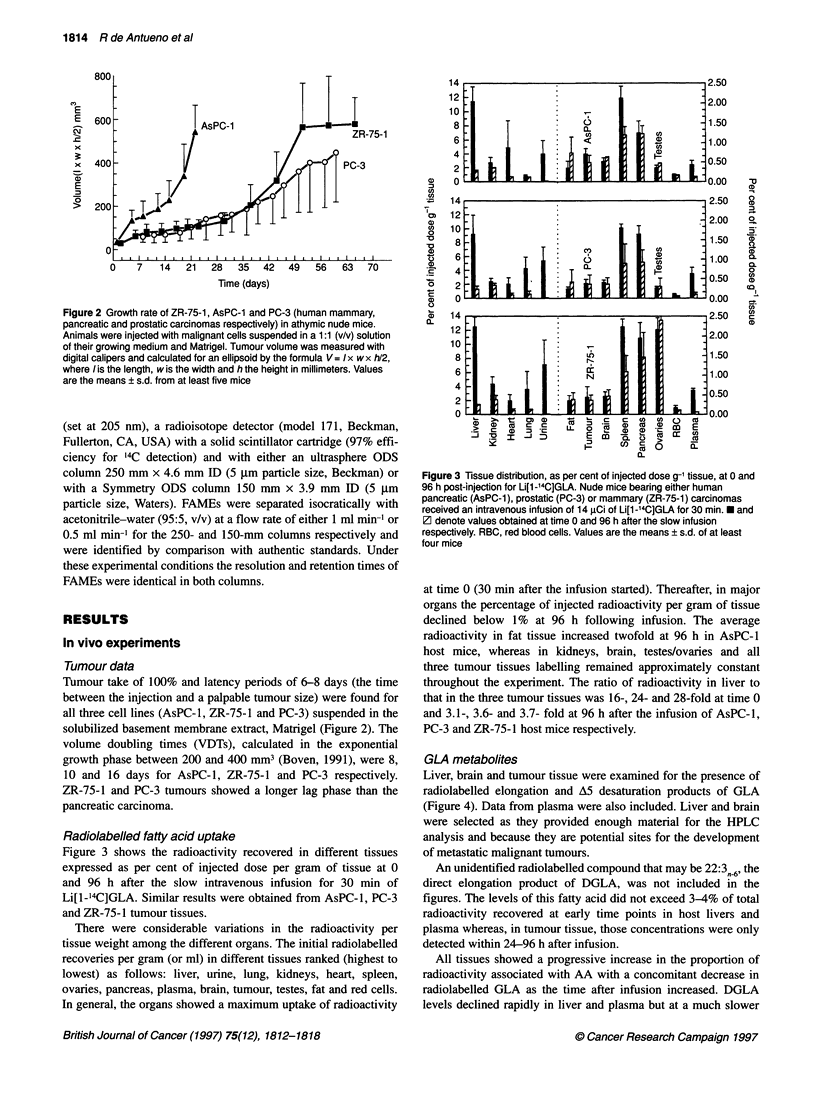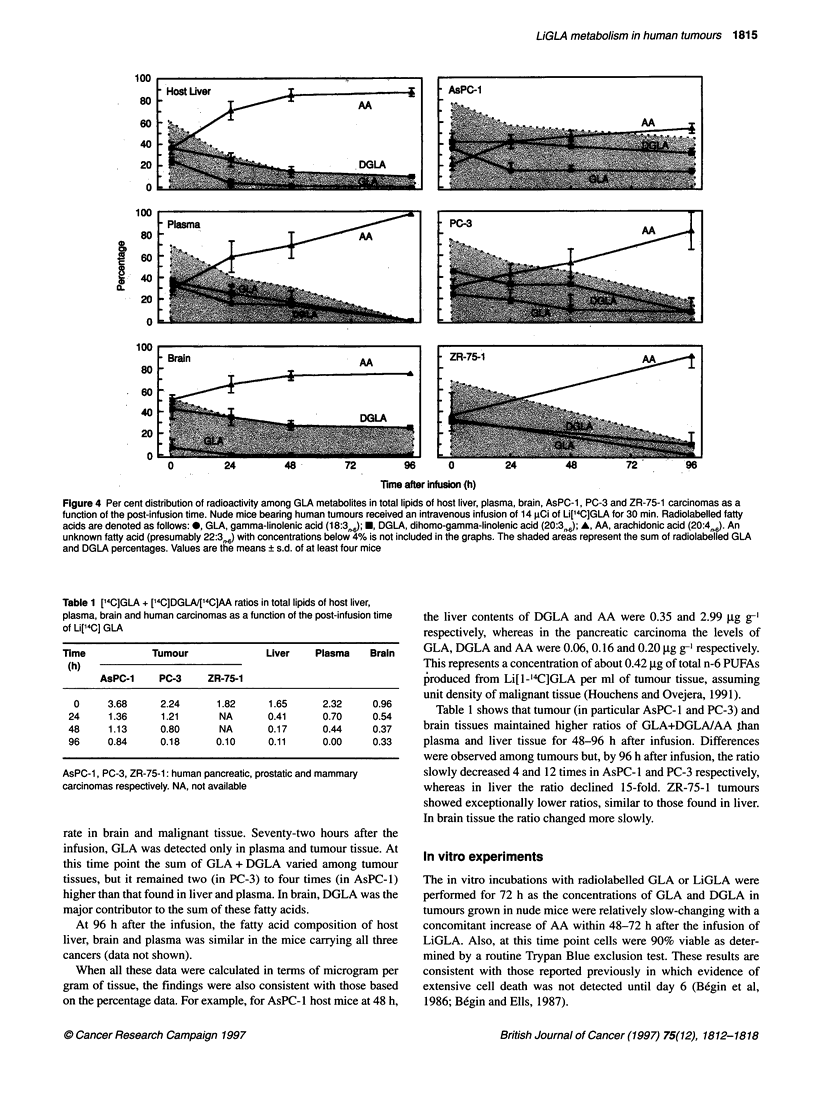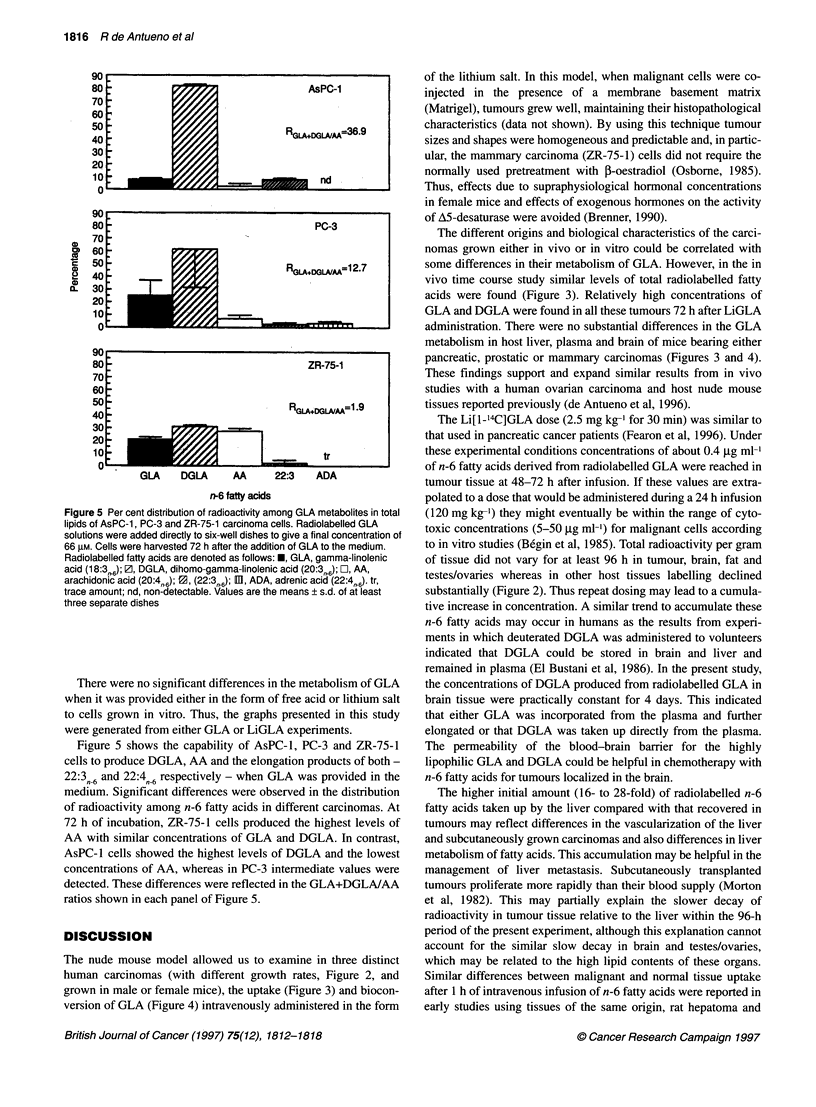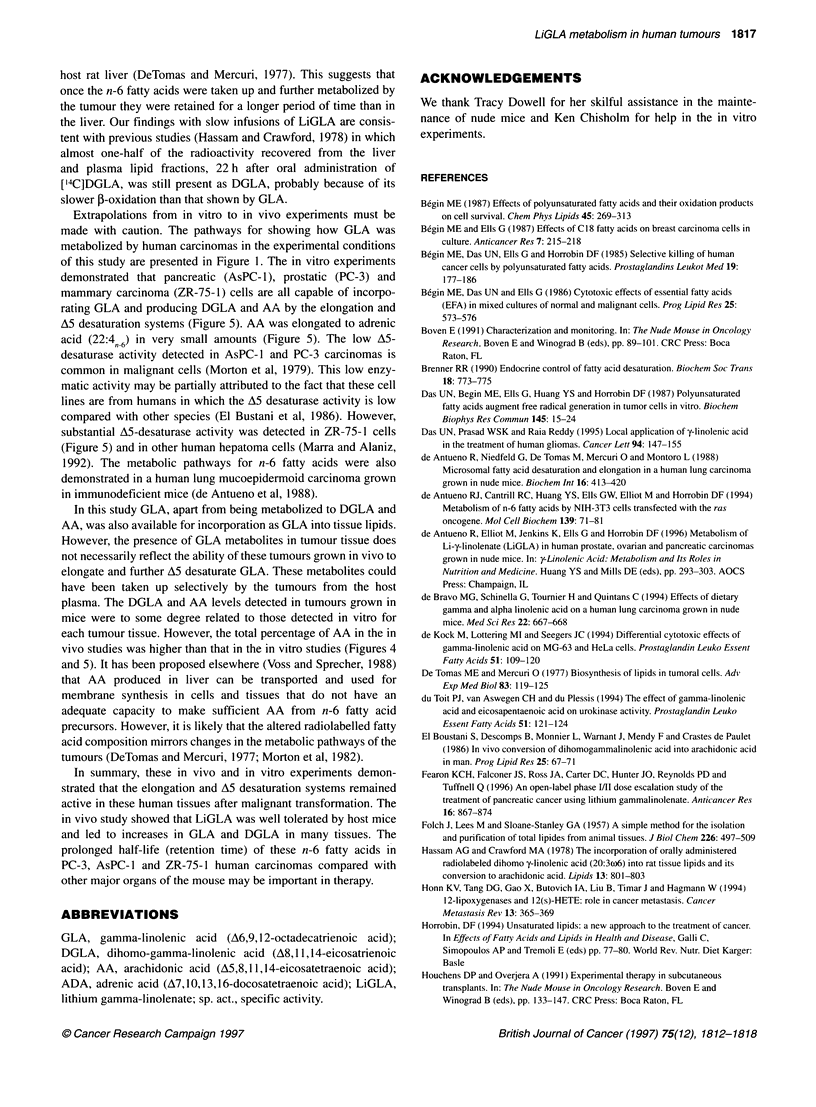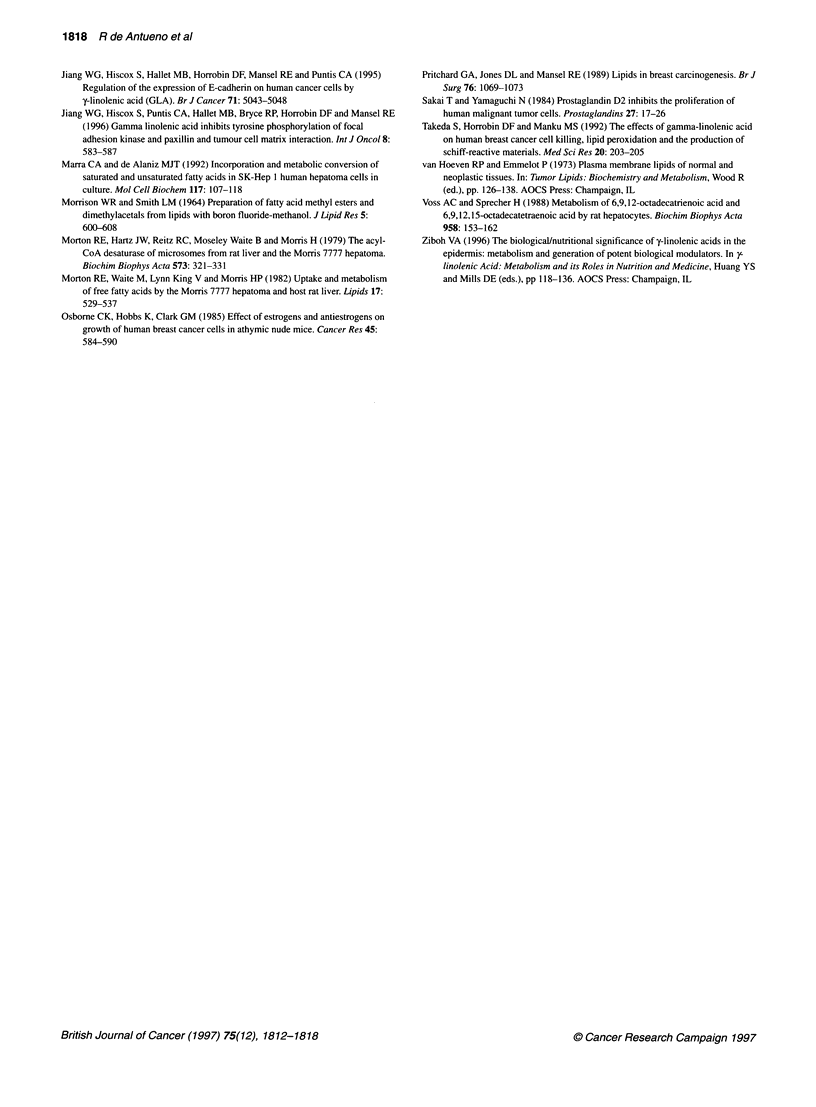# In vivo and in vitro biotransformation of the lithium salt of gamma-linolenic acid by three human carcinomas.

**DOI:** 10.1038/bjc.1997.309

**Published:** 1997

**Authors:** R. de Antueno, M. Elliot, G. Ells, P. Quiroga, K. Jenkins, D. Horrobin

**Affiliations:** Scotia Research Institute, Kentville, NS, Canada.

## Abstract

Lipid metabolism has been considered recently as a novel target for cancer therapy. In this field, lithium gamma-linolenate (LiGLA) is a promising experimental compound for use in the treatment of human tumours. In vivo and in vitro studies allowed us to assess the metabolism of radiolabelled LiGLA by tumour tissue and different organs of the host. In vitro studies demonstrated that human pancreatic (AsPC-1), prostatic (PC-3) and mammary carcinoma (ZR-75-1) cells were capable of elongating GLA from LiGLA to dihomo-gamma-linolenic acid (DGLA) and further desaturating it to arachidonic acid (AA). AsPC-1 cells showed the lowest delta5-desaturase activity on DGLA. In the in vivo studies, nude mice bearing the human carcinomas were given Li[1-(14)C]GLA (2.5 mg kg(-1)) by intravenous injection for 30 min. Mice were either sacrificed after infusion or left for up to 96 h recovery before sacrifice. In general, the organs showed a maximum uptake of radioactivity 30 min after the infusion started (t = 0). Thereafter, in major organs the percentage of injected radioactivity per g of tissue declined below 1% 96 h after infusion. In kidney, brain, testes/ovaries and all three tumour tissues, labelling remained constant throughout the experiment. The ratio of radioactivity in liver to tumour tissues ranged between 16- and 24-fold at t = 0 and between 3.1- and 3.7-fold at 96 h. All tissues showed a progressive increase in the proportion of radioactivity associated with AA with a concomitant decrease in radiolabelled GLA as the time after infusion increased. DGLA declined rapidly in liver and plasma, but at a much slower rate in brain and malignant tissue. Seventy-two hours after the infusion, GLA was only detected in plasma and tumour tissue. The sum of GLA + DGLA varied among tumour tissues, but it remained 2-4 times higher than in liver and plasma. In brain, DGLA is the major contributor to the sum of these fatty acids. Data showed that cytotoxic GLA and DGLA, the latter provided either by the host or by endogenous synthesis, remained in human tumours for at least 4 days.


British Journal of Cancer (1997) 75(12), 1812–1818
© 1997 Cancer Research Campaign

### In vivo and in vitro biotransformation of the lithium salt of gamma-linolenic acid by three human carcinomas

**R de Antueno, M Elliot, G Ells, P Quiroga, K Jenkins and D Horrobin**

Scotia Research Institute, PO Box 818, Kentville, NS, Canada, B4N 4H8

**Summary** Lipid metabolism has been considered recently as a novel target for cancer therapy. In this field, lithium gamma-linolenate (LiGLA) is a promising experimental compound for use in the treatment of human tumours. In vivo and in vitro studies allowed us to assess the metabolism of radiolabelled LiGLA by tumour tissue and different organs of the host. In vitro studies demonstrated that human pancreatic (AsPC-1), prostatic (PC-3) and mammary carcinoma (ZR-75-1) cells were capable of elongating GLA from LiGLA to dihomo-gamma-linolenic acid (DGLA) and further desaturating it to arachidonic acid (AA). AsPC-1 cells showed the lowest Δ5-desaturase activity on DGLA. In the in vivo studies, nude mice bearing the human carcinomas were given Li[1-$^{14}$C]GLA (2.5 mg kg$^{-1}$) by intravenous injection for 30 min. Mice were either sacrificed after infusion or left for up to 96 h recovery before sacrifice. In general, the organs showed a maximum uptake of radioactivity 30 min after the infusion started ($t = 0$). Thereafter, in major organs the percentage of injected radioactivity per g of tissue declined below 1% 96 h after infusion. In kidney, brain, testes/ovaries and all three tumour tissues, labelling remained constant throughout the experiment. The ratio of radioactivity in liver to tumour tissues ranged between 16- and 24-fold at $t = 0$ and between 3.1- and 3.7-fold at 96 h. All tissues showed a progressive increase in the proportion of radioactivity associated with AA with a concomitant decrease in radiolabelled GLA as the time after infusion increased. DGLA declined rapidly in liver and plasma, but at a much slower rate in brain and malignant tissue. Seventy-two hours after the infusion, GLA was only detected in plasma and tumour tissue. The sum of GLA + DGLA varied among tumour tissues, but it remained 2–4 times higher than in liver and plasma. In brain, DGLA is the major contributor to the sum of these fatty acids. Data showed that cytotoxic GLA and DGLA, the latter provided either by the host or by endogenous synthesis, remained in human tumours for at least 4 days.

**Keywords:** lithium gamma-linolenate; gamma-linolenic acid; AsPC-1; ZR-75-1; PC-3; nude mice; dihomo-gamma-linolenic acid

### INTRODUCTION

Gamma-linolenic acid (GLA; $18{:}3_{n\text{-}6}$) and dihomo-gamma-linolenic acid (DGLA $20{:}3_{n\text{-}6}$), both with three double bonds in the *cis* configuration, have been shown to have a wide range of anti-cancer and/or antiproliferative effects (Horrobin, 1994; Jiang et al, 1995). These *n*-6 fatty acids are derived from linolenic acid ($18{:}2_{n\text{-}6}$) and can be converted to arachidonic acid (AA; $20{:}4_{n\text{-}6}$), as shown in Figure 1.

Co-culture experiments in which normal and malignant cells were cultured together in the same dish demonstrated that cancer cells were selectively responsive to GLA and its elongation product, DGLA, when added to the culture medium at concentrations that do not harm normal cells (Bégin et al, 1986). In contrast, arachidonic acid, the Δ5 desaturation product of DGLA, exhibits cytotoxic effects on cancer cells but is not as selective as its precursors (Bégin, 1987).

One possible mechanism of action is based on the fact that these *n*-6 fatty acids can bypass the Δ6-desaturase deficiency reported in some malignant cells and/or restore towards normal the low levels of polyunsaturated fatty acids detected in certain cancer patients (van Hoeven and Emmelot 1973; Feraon et al, 1996). On exposure to GLA the cancer cells generate higher levels of superoxide radicals, lipid peroxidation products and/or eiscosanoids, many of which compounds have anti-tumour properties (Sakai and Yamaguchi, 1984; Das et al, 1987; Takeda et al, 1992).

Furthermore, GLA seems to have the ability to induce apoptosis (de Kock et al, 1994) and to inhibit a range of mechanisms responsible for metastasis. Indeed, GLA has now been shown to restore defective E-cadherin function in human lung, colon, breast, melanoma and liver cancer cells, with a corresponding loss of invasiveness (Jiang et al, 1995). GLA or its metabolites were

**Figure 1** GLA elongation and desaturation pathways. Abbreviations: GLA, gamma-linolenic acid ($18{:}3_{n\text{-}6}$); DGLA, dihomo-gamma-linolenic acid ($20{:}3_{n\text{-}6}$); AA, arachidonic acid ($20{:}4_{n\text{-}6}$); $22{:}3_{n\text{-}6}$, direct elongation product of DGLA; ADA, adrenic acid ($22{:}4_{n\text{-}6}$)

*Received 26 September 1996*
*Revised 9 December 1996*
*Accepted 18 December 1996*

*Correspondence to:* R de Antueno, Scotia Research Institute, PO Box 818, Kentville, NS, Canada, B4N 4H8

effective in inhibiting enzymes associated with metastasis or closely correlated with metastatic potential such as urokinase (du Toit et al, 1994), 12-lipoxygenase (Honn et al, 1994; Ziboh, 1996) and 5-lipoxygenase (Ziboh, 1996). In addition, GLA inhibits cell–matrix interactions and so GLA exerts inhibitory effects at several stages in the multistep process of tumour formation and progression (Jiang et al, 1996).

Particularly promising therapeutic effects were obtained with the administration of GLA directly into the tumour cavity in patients with malignant glioblastomas and astrocytomas (Das et al, 1995). The tumours regressed and survival was substantially prolonged. The inhibitory effect of GLA on the growth of a human lung mucoepidermoid carcinoma, breast cancer and malignant melanoma (de Bravo et al, 1994; Pritchard et al, 1989) was also observed in immunodeficient animal models.

In most of these studies GLA was either administered orally in the form of triglyceride, ester or free fatty acid or injected intravenously in the form of the relatively water-soluble lithium salt (Fearon et al, 1996). Potassium salts were not considered because of potentially adverse cardiac effects. The lithium salt had an advantage over the sodium salt in that the rate of infusion could be monitored by measuring the lithium blood concentrations with routine hospital analytical techniques. Toxicity of lithium is not a concern if the serum levels of lithium remain below those commonly used in chronic psychiatric treatments (Fearon et al, 1996). A phase I/II dose escalation study in patients with inoperable pancreatic cancer showed that the higher doses of LiGLA were associated with longer survival times without an important adverse outcome and, in particular, without the serious effects commonly related with chemotherapy (Fearon et al, 1996).

As unsaturated lipids are now serious candidates for novel anticancer drugs, it is important to understand the metabolism of LiGLA in cancer patients. We have therefore examined the in vivo and in vitro uptake, elongation and further desaturation of radiolabelled LiGLA by three human carcinomas.

### MATERIALS AND METHODS

### Animals and human carcinomas

Seven-week-old athymic CD1BR (nu/nu) mice were obtained from Charles River Canada (St Constant, Quebec, Canada) and housed in polycarbonate cages with air filter tops in a pathogen temperature-controlled environment (25 ± 1°C) with a 12-h light/dark cycle. Animals were maintained ad libitum on a gamma-irradiated chow diet and sterile water (pH 2.5). Mouse manipulations were performed in a class II laminar flow hood.

ZR-75-1, PC-3 and AsPC-1 (human mammary, prostatic and pancreatic carcinomas respectively; American Type Culture Collection, Rockville, MD, USA) were grown in Dulbecco's modification of Eagle medium [DMEM; containing 10% fetal bovine serum (FBS), penicillin (50 IU $ml^{-1}$) and streptomycin (50 μg $ml^{-1}$)] in a humidified atmosphere of 95% air and 5% carbon dioxide at 37°C. Cell culture media and supplements were obtained from ICN Biomedicals, Costa Mesa, CA, USA.

### In vitro experiments

At 90% confluency, malignant cells were incubated in fresh medium with either the free acid form or the lithium salt of [1-$^{14}$C]GLA (sp. act. 58 mCi $mmol^{-1}$; New England Nuclear, Boston, MA, USA). Radiolabelled GLA or LiGLA was diluted with unlabelled fatty acids (Nu-Check Prep, Elysian, MN, USA) to give a final specific activity of 6.06 mCi $mmol^{-1}$. Ethanolic fatty acid solutions were added directly to six-well dishes to give a final concentration of 66 μM and 0.1% for fatty acids and ethanol respectively (total radioactivity 1 μCi in 2.5 ml). Three days after the addition of fatty acids, the cell monolayer was washed three times with phosphate-buffered saline (PBS) and the cells were harvested in PBS using a rubber policeman. The cell suspension was kept in the freezer (–20°C) until lipid extraction.

### In vivo experiments

All cell lines were suspended in their growth medium and a solubilized attachment matrix, Matrigel (1/1 v/v; Collaborative Research, Bedford, MA, USA). Male mice were injected s.c. into the interscapular region with 300 μl of the suspension containing $5 \times 10^6$ of either AsPC-1 or PC-3 cells, whereas female mice were injected with the same amount of ZR-75-1 cells.

When tumours became palpable, they were measured twice weekly with digital slide calipers (FV Fowler, Newton, MA, USA) connected to a computerized system. Tumour volume was calculated for an ellipsoid by the formula $V = l \times w \times h/2$, where $l$ is the length, $w$ is the width and $h$ is the height, in millimetres.

### Li[1-$^{14}$C]GLA administration

In the in vivo experiments conditions were designed to mimic, as much as is possible in an animal model, the infusion protocol followed with cancer patients (Fearon et al, 1996).

At approximately 300 $mm^3$ tumour volume animals were anaesthetized with sodium pentobarbital (80 mg $kg^{-1}$; Somnotol, MTC Pharmaceuticals, Cambridge, Ontario, Canada). Mice were then given 125 μl of 0.9% sodium chloride with 200 IU $kg^{-1}$ heparin and 14 μCi of Li[1-$^{14}$C]GLA (more than 98% radiochemical purity; 58 μCi $\mu mol^{-1}$; New England Nuclear) by slow intravenous injection through the tail vein (2.53 mg $kg^{-1}$ over 30 min) using a paediatric 27 G butterfly and infusion pump.

Mice were sacrificed either immediately after the 30 min infusion (time 0) or left for recovery for either 24, 48 or 96 h until sacrifice by exsanguination under halothane anaesthesia.

Organs were perfused (via the heart left ventricle, vena cava and portal vein) and rinsed with cold 0.9% sodium chloride and blotted dry. Aliquots of each tissue were solubilized with Solvable 0.5 M (New England Nuclear) at 56°C.

In order to reduce chemiluminescence, samples were decolorized with hydrogen peroxide and neutralized with glacial acetic acid before adding the cocktail (Formula 989, New England Nuclear) for scintillation counting.

### Lipid analysis

Lipids from malignant cell suspensions and from organ and tumour tissues were extracted with chloroform–methanol (2:1, v/v) (Folch et al, 1957). The total lipid extract was methylated using boron fluoride in methanol at 90°C for 30 min (Morrison and Smith, 1964). The resultant radiolabelled fatty acid methyl esters (FAMEs) were analysed as previously described (de Antueno et al, 1994). Analyses were carried out on a Waters Associates (Milford, MA, USA) high-performance liquid chromatograph equipped with a variable wavelength UV–VIS monitor

© Cancer Research Campaign 1997

**Figure 2** Growth rate of ZR-75-1, AsPC-1 and PC-3 (human mammary, pancreatic and prostatic carcinomas respectively) in athymic nude mice. Animals were injected with malignant cells suspended in a 1:1 (v/v) solution of their growing medium and Matrigel. Tumour volume was measured with digital calipers and calculated for an ellipsoid by the formula $V = l \times w \times h/2$, where $l$ is the length, $w$ is the width and $h$ the height in millimeters. Values are the means ± s.d. from at least five mice

**Figure 3** Tissue distribution, as per cent of injected dose g$^{-1}$ tissue, at 0 and 96 h post-injection for Li[1-$^{14}$C]GLA. Nude mice bearing either human pancreatic (AsPC-1), prostatic (PC-3) or mammary (ZR-75-1) carcinomas received an intravenous infusion of 14 µCi of Li[1-$^{14}$C]GLA for 30 min. ■ and ▨ denote values obtained at time 0 and 96 h after the slow infusion respectively. RBC, red blood cells. Values are the means ± s.d. of at least four mice

(set at 205 nm), a radioisotope detector (model 171, Beckman, Fullerton, CA, USA) with a solid scintillator cartridge (97% efficiency for $^{14}$C detection) and with either an ultrasphere ODS column 250 mm × 4.6 mm ID (5 µm particle size, Beckman) or with a Symmetry ODS column 150 mm × 3.9 mm ID (5 µm particle size, Waters). FAMEs were separated isocratically with acetonitrile–water (95:5, v/v) at a flow rate of either 1 ml min$^{-1}$ or 0.5 ml min$^{-1}$ for the 250- and 150-mm columns respectively and were identified by comparison with authentic standards. Under these experimental conditions the resolution and retention times of FAMEs were identical in both columns.

### RESULTS

### In vivo experiments

#### *Tumour data*

Tumour take of 100% and latency periods of 6–8 days (the time between the injection and a palpable tumour size) were found for all three cell lines (AsPC-1, ZR-75-1 and PC-3) suspended in the solubilized basement membrane extract, Matrigel (Figure 2). The volume doubling times (VDTs), calculated in the exponential growth phase between 200 and 400 mm$^3$ (Boven, 1991), were 8, 10 and 16 days for AsPC-1, ZR-75-1 and PC-3 respectively. ZR-75-1 and PC-3 tumours showed a longer lag phase than the pancreatic carcinoma.

#### *Radiolabelled fatty acid uptake*

Figure 3 shows the radioactivity recovered in different tissues expressed as per cent of injected dose per gram of tissue at 0 and 96 h after the slow intravenous infusion for 30 min of Li[1-$^{14}$C]GLA. Similar results were obtained from AsPC-1, PC-3 and ZR-75-1 tumour tissues.

There were considerable variations in the radioactivity per tissue weight among the different organs. The initial radiolabelled recoveries per gram (or ml) in different tissues ranked (highest to lowest) as follows: liver, urine, lung, kidneys, heart, spleen, ovaries, pancreas, plasma, brain, tumour, testes, fat and red cells. In general, the organs showed a maximum uptake of radioactivity at time 0 (30 min after the infusion started). Thereafter, in major organs the percentage of injected radioactivity per gram of tissue declined below 1% at 96 h following infusion. The average radioactivity in fat tissue increased twofold at 96 h in AsPC-1 host mice, whereas in kidneys, brain, testes/ovaries and all three tumour tissues labelling remained approximately constant throughout the experiment. The ratio of radioactivity in liver to that in the three tumour tissues was 16-, 24- and 28-fold at time 0 and 3.1-, 3.6- and 3.7- fold at 96 h after the infusion of AsPC-1, PC-3 and ZR-75-1 host mice respectively.

#### *GLA metabolites*

Liver, brain and tumour tissue were examined for the presence of radiolabelled elongation and Δ5 desaturation products of GLA (Figure 4). Data from plasma were also included. Liver and brain were selected as they provided enough material for the HPLC analysis and because they are potential sites for the development of metastatic malignant tumours.

An unidentified radiolabelled compound that may be $22{:}3_{n\text{-}6}$, the direct elongation product of DGLA, was not included in the figures. The levels of this fatty acid did not exceed 3–4% of total radioactivity recovered at early time points in host livers and plasma whereas, in tumour tissue, those concentrations were only detected within 24–96 h after infusion.

All tissues showed a progressive increase in the proportion of radioactivity associated with AA with a concomitant decrease in radiolabelled GLA as the time after infusion increased. DGLA levels declined rapidly in liver and plasma but at a much slower

© Cancer Research Campaign 1997

**Figure 4** Per cent distribution of radioactivity among GLA metabolites in total lipids of host liver, plasma, brain, AsPC-1, PC-3 and ZR-75-1 carcinomas as a function of the post-infusion time. Nude mice bearing human tumours received an intravenous infusion of 14 μCi of Li[$^{14}$C]GLA for 30 min. Radiolabelled fatty acids are denoted as follows: ●, GLA, gamma-linolenic acid ($18{:}3_{n\text{-}6}$); ■, DGLA, dihomo-gamma-linolenic acid ($20{:}3_{n\text{-}6}$); ▲, AA, arachidonic acid ($20{:}4_{n\text{-}6}$). An unknown fatty acid (presumably $22{:}3_{n\text{-}6}$) with concentrations below 4% is not included in the graphs. The shaded areas represent the sum of radiolabelled GLA and DGLA percentages. Values are the means ± s.d. of at least four mice

**Table 1** [$^{14}$C]GLA + [$^{14}$C]DGLA/[$^{14}$C]AA ratios in total lipids of host liver, plasma, brain and human carcinomas as a function of the post-infusion time of Li[$^{14}$C] GLA

| Time (h) | Tumour | | | Liver | Plasma | Brain |
|---|---|---|---|---|---|---|
| | AsPC-1 | PC-3 | ZR-75-1 | | | |
| 0 | 3.68 | 2.24 | 1.82 | 1.65 | 2.32 | 0.96 |
| 24 | 1.36 | 1.21 | NA | 0.41 | 0.70 | 0.54 |
| 48 | 1.13 | 0.80 | NA | 0.17 | 0.44 | 0.37 |
| 96 | 0.84 | 0.18 | 0.10 | 0.11 | 0.00 | 0.33 |

AsPC-1, PC-3, ZR-75-1: human pancreatic, prostatic and mammary carcinomas respectively. NA, not available rate in brain and malignant tissue. Seventy-two hours after the infusion, GLA was detected only in plasma and tumour tissue. At this time point the sum of GLA + DGLA varied among tumour tissues, but it remained two (in PC-3) to four times (in AsPC-1) higher than that found in liver and plasma. In brain, DGLA was the major contributor to the sum of these fatty acids.

At 96 h after the infusion, the fatty acid composition of host liver, brain and plasma was similar in the mice carrying all three cancers (data not shown).

When all these data were calculated in terms of microgram per gram of tissue, the findings were also consistent with those based on the percentage data. For example, for AsPC-1 host mice at 48 h, the liver contents of DGLA and AA were 0.35 and 2.99 μg $g^{-1}$ respectively, whereas in the pancreatic carcinoma the levels of GLA, DGLA and AA were 0.06, 0.16 and 0.20 μg $g^{-1}$ respectively. This represents a concentration of about 0.42 μg of total n-6 PUFAs produced from Li[1-$^{14}$C]GLA per ml of tumour tissue, assuming unit density of malignant tissue (Houchens and Ovejera, 1991).

Table 1 shows that tumour (in particular AsPC-1 and PC-3) and brain tissues maintained higher ratios of GLA+DGLA/AA than plasma and liver tissue for 48–96 h after infusion. Differences were observed among tumours but, by 96 h after infusion, the ratio slowly decreased 4 and 12 times in AsPC-1 and PC-3 respectively, whereas in liver the ratio declined 15-fold. ZR-75-1 tumours showed exceptionally lower ratios, similar to those found in liver. In brain tissue the ratio changed more slowly.

### In vitro experiments

The in vitro incubations with radiolabelled GLA or LiGLA were performed for 72 h as the concentrations of GLA and DGLA in tumours grown in nude mice were relatively slow-changing with a concomitant increase of AA within 48–72 h after the infusion of LiGLA. Also, at this time point cells were 90% viable as determined by a routine Trypan Blue exclusion test. These results are consistent with those reported previously in which evidence of extensive cell death was not detected until day 6 (Bégin et al, 1986; Bégin and Ells, 1987).

**Figure 5** Per cent distribution of radioactivity among GLA metabolites in total lipids of AsPC-1, PC-3 and ZR-75-1 carcinoma cells. Radiolabelled GLA solutions were added directly to six-well dishes to give a final concentration of 66 μM. Cells were harvested 72 h after the addition of GLA to the medium. Radiolabelled fatty acids are denoted as follows: ■, GLA, gamma-linolenic acid ($18:3_{n\text{-}6}$); ▨, DGLA, dihomo-gamma-linolenic acid ($20:3_{n\text{-}6}$); □, AA, arachidonic acid ($20:4_{n\text{-}6}$); ▨, ($22:3_{n\text{-}6}$); ▥, ADA, adrenic acid ($22:4_{n\text{-}6}$). tr, trace amount; nd, non-detectable. Values are the means ± s.d. of at least three separate dishes

There were no significant differences in the metabolism of GLA when it was provided either in the form of free acid or lithium salt to cells grown in vitro. Thus, the graphs presented in this study were generated from either GLA or LiGLA experiments.

Figure 5 shows the capability of AsPC-1, PC-3 and ZR-75-1 cells to produce DGLA, AA and the elongation products of both – $22:3_{n\text{-}6}$ and $22:4_{n\text{-}6}$ respectively – when GLA was provided in the medium. Significant differences were observed in the distribution of radioactivity among *n*-6 fatty acids in different carcinomas. At 72 h of incubation, ZR-75-1 cells produced the highest levels of AA with similar concentrations of GLA and DGLA. In contrast, AsPC-1 cells showed the highest levels of DGLA and the lowest concentrations of AA, whereas in PC-3 intermediate values were detected. These differences were reflected in the GLA+DGLA/AA ratios shown in each panel of Figure 5.

### DISCUSSION

The nude mouse model allowed us to examine in three distinct human carcinomas (with different growth rates, Figure 2, and grown in male or female mice), the uptake (Figure 3) and bioconversion of GLA (Figure 4) intravenously administered in the form of the lithium salt. In this model, when malignant cells were co-injected in the presence of a membrane basement matrix (Matrigel), tumours grew well, maintaining their histopathological characteristics (data not shown). By using this technique tumour sizes and shapes were homogeneous and predictable and, in particular, the mammary carcinoma (ZR-75-1) cells did not require the normally used pretreatment with β-oestradiol (Osborne, 1985). Thus, effects due to supraphysiological hormonal concentrations in female mice and effects of exogenous hormones on the activity of Δ5-desaturase were avoided (Brenner, 1990).

The different origins and biological characteristics of the carcinomas grown either in vivo or in vitro could be correlated with some differences in their metabolism of GLA. However, in the in vivo time course study similar levels of total radiolabelled fatty acids were found (Figure 3). Relatively high concentrations of GLA and DGLA were found in all these tumours 72 h after LiGLA administration. There were no substantial differences in the GLA metabolism in host liver, plasma and brain of mice bearing either pancreatic, prostatic or mammary carcinomas (Figures 3 and 4). These findings support and expand similar results from in vivo studies with a human ovarian carcinoma and host nude mouse tissues reported previously (de Antueno et al, 1996).

The Li[1-$^{14}$C]GLA dose (2.5 mg $kg^{-1}$ for 30 min) was similar to that used in pancreatic cancer patients (Fearon et al, 1996). Under these experimental conditions concentrations of about 0.4 μg $ml^{-1}$ of *n*-6 fatty acids derived from radiolabelled GLA were reached in tumour tissue at 48–72 h after infusion. If these values are extrapolated to a dose that would be administered during a 24 h infusion (120 mg $kg^{-1}$) they might eventually be within the range of cytotoxic concentrations (5–50 μg $ml^{-1}$) for malignant cells according to in vitro studies (Bégin et al, 1985). Total radioactivity per gram of tissue did not vary for at least 96 h in tumour, brain, fat and testes/ovaries whereas in other host tissues labelling declined substantially (Figure 2). Thus repeat dosing may lead to a cumulative increase in concentration. A similar trend to accumulate these *n*-6 fatty acids may occur in humans as the results from experiments in which deuterated DGLA was administered to volunteers indicated that DGLA could be stored in brain and liver and remained in plasma (El Bustani et al, 1986). In the present study, the concentrations of DGLA produced from radiolabelled GLA in brain tissue were practically constant for 4 days. This indicated that either GLA was incorporated from the plasma and further elongated or that DGLA was taken up directly from the plasma. The permeability of the blood–brain barrier for the highly lipophilic GLA and DGLA could be helpful in chemotherapy with *n*-6 fatty acids for tumours localized in the brain.

The higher initial amount (16- to 28-fold) of radiolabelled *n*-6 fatty acids taken up by the liver compared with that recovered in tumours may reflect differences in the vascularization of the liver and subcutaneously grown carcinomas and also differences in liver metabolism of fatty acids. This accumulation may be helpful in the management of liver metastasis. Subcutaneously transplanted tumours proliferate more rapidly than their blood supply (Morton et al, 1982). This may partially explain the slower decay of radioactivity in tumour tissue relative to the liver within the 96-h period of the present experiment, although this explanation cannot account for the similar slow decay in brain and testes/ovaries, which may be related to the high lipid contents of these organs. Similar differences between malignant and normal tissue uptake after 1 h of intravenous infusion of *n*-6 fatty acids were reported in early studies using tissues of the same origin, rat hepatoma and

 © Cancer Research Campaign 1997

host rat liver (DeTomas and Mercuri, 1977). This suggests that once the *n*-6 fatty acids were taken up and further metabolized by the tumour they were retained for a longer period of time than in the liver. Our findings with slow infusions of LiGLA are consistent with previous studies (Hassam and Crawford, 1978) in which almost one-half of the radioactivity recovered from the liver and plasma lipid fractions, 22 h after oral administration of [$^{14}$C]DGLA, was still present as DGLA, probably because of its slower β-oxidation than that shown by GLA.

Extrapolations from in vitro to in vivo experiments must be made with caution. The pathways for showing how GLA was metabolized by human carcinomas in the experimental conditions of this study are presented in Figure 1. The in vitro experiments demonstrated that pancreatic (AsPC-1), prostatic (PC-3) and mammary carcinoma (ZR-75-1) cells are all capable of incorporating GLA and producing DGLA and AA by the elongation and Δ5 desaturation systems (Figure 5). AA was elongated to adrenic acid ($22{:}4_{n\text{-}6}$) in very small amounts (Figure 5). The low Δ5-desaturase activity detected in AsPC-1 and PC-3 carcinomas is common in malignant cells (Morton et al, 1979). This low enzymatic activity may be partially attributed to the fact that these cell lines are from humans in which the Δ5 desaturase activity is low compared with other species (El Bustani et al, 1986). However, substantial Δ5-desaturase activity was detected in ZR-75-1 cells (Figure 5) and in other human hepatoma cells (Marra and Alaniz, 1992). The metabolic pathways for *n*-6 fatty acids were also demonstrated in a human lung mucoepidermoid carcinoma grown in immunodeficient mice (de Antueno et al, 1988).

In this study GLA, apart from being metabolized to DGLA and AA, was also available for incorporation as GLA into tissue lipids. However, the presence of GLA metabolites in tumour tissue does not necessarily reflect the ability of these tumours grown in vivo to elongate and further Δ5 desaturate GLA. These metabolites could have been taken up selectively by the tumours from the host plasma. The DGLA and AA levels detected in tumours grown in mice were to some degree related to those detected in vitro for each tumour tissue. However, the total percentage of AA in the in vivo studies was higher than that in the in vitro studies (Figures 4 and 5). It has been proposed elsewhere (Voss and Sprecher, 1988) that AA produced in liver can be transported and used for membrane synthesis in cells and tissues that do not have an adequate capacity to make sufficient AA from *n*-6 fatty acid precursors. However, it is likely that the altered radiolabelled fatty acid composition mirrors changes in the metabolic pathways of the tumours (DeTomas and Mercuri, 1977; Morton et al, 1982).

In summary, these in vivo and in vitro experiments demonstrated that the elongation and Δ5 desaturation systems remained active in these human tissues after malignant transformation. The in vivo study showed that LiGLA was well tolerated by host mice and led to increases in GLA and DGLA in many tissues. The prolonged half-life (retention time) of these *n*-6 fatty acids in PC-3, AsPC-1 and ZR-75-1 human carcinomas compared with other major organs of the mouse may be important in therapy.

### ABBREVIATIONS

GLA, gamma-linolenic acid (Δ6,9,12-octadecatrienoic acid); DGLA, dihomo-gamma-linolenic acid (Δ8,11,14-eicosatrienoic acid); AA, arachidonic acid (Δ5,8,11,14-eicosatetraenoic acid); ADA, adrenic acid (Δ7,10,13,16-docosatetraenoic acid); LiGLA, lithium gamma-linolenate; sp. act., specific activity.

### ACKNOWLEDGEMENTS

We thank Tracy Dowell for her skilful assistance in the maintenance of nude mice and Ken Chisholm for help in the in vitro experiments.

### REFERENCES

Bégin ME (1987) Effects of polyunsaturated fatty acids and their oxidation products on cell survival. *Chem Phys Lipids* **45**: 269–313

Bégin ME and Ells G (1987) Effects of C18 fatty acids on breast carcinoma cells in culture. *Anticancer Res* **7**: 215–218

Bégin ME, Das UN, Ells G and Horrobin DF (1985) Selective killing of human cancer cells by polyunsaturated fatty acids. *Prostaglandins Leukot Med* **19**: 177–186

Bégin ME, Das UN and Ells G (1986) Cytotoxic effects of essential fatty acids (EFA) in mixed cultures of normal and malignant cells. *Prog Lipid Res* **25**: 573–576

Boven E (1991) Characterization and monitoring. In: *The Nude Mouse in Oncology Research*, Boven E and Winograd B (eds), pp. 89–101. CRC Press: Boca Raton, FL

Brenner RR (1990) Endocrine control of fatty acid desaturation. *Biochem Soc Trans* **18**: 773–775

Das UN, Begin ME, Ells G, Huang YS and Horrobin DF (1987) Polyunsaturated fatty acids augment free radical generation in tumor cells in vitro. *Biochem Biophys Res Commun* **145**: 15–24

Das UN, Prasad WSK and Raia Reddy (1995) Local application of γ-linolenic acid in the treatment of human gliomas. *Cancer Lett* **94**: 147–155

de Antueno R, Niedfeld G, De Tomas M, Mercuri O and Montoro L (1988) Microsomal fatty acid desaturation and elongation in a human lung carcinoma grown in nude mice. *Biochem Int* **16**: 413–420

de Antueno RJ, Cantrill RC, Huang YS, Ells GW, Elliot M and Horrobin DF (1994) Metabolism of n-6 fatty acids by NIH-3T3 cells transfected with the *ras* oncogene. *Mol Cell Biochem* **139**: 71–81

de Antueno R, Elliot M, Jenkins K, Ells G and Horrobin DF (1996) Metabolism of Li-γ-linolenate (LiGLA) in human prostate, ovarian and pancreatic carcinomas grown in nude mice. In: *γ-Linolenic Acid: Metabolism and Its Roles in Nutrition and Medicine*. Huang YS and Mills DE (eds), pp. 293–303. AOCS Press: Champaign, IL

de Bravo MG, Schinella G, Tournier H and Quintans C (1994) Effects of dietary gamma and alpha linolenic acid on a human lung carcinoma grown in nude mice. *Med Sci Res* **22**: 667–668

de Kock M, Lottering MI and Seegers JC (1994) Differential cytotoxic effects of gamma-linolenic acid on MG-63 and HeLa cells. *Prostaglandin Leuko Essent Fatty Acids* **51**: 109–120

De Tomas ME and Mercuri O (1977) Biosynthesis of lipids in tumoral cells. *Adv Exp Med Biol* **83**: 119–125

du Toit PJ, van Aswegen CH and du Plessis (1994) The effect of gamma-linolenic acid and eicosapentaenoic acid on urokinase activity. *Prostaglandin Leuko Essent Fatty Acids* **51**: 121–124

El Boustani S, Descomps B, Monnier L, Warnant J, Mendy F and Crastes de Paulet (1986) In vivo conversion of dihomogammalinolenic acid into arachidonic acid in man. *Prog Lipid Res* **25**: 67–71

Fearon KCH, Falconer JS, Ross JA, Carter DC, Hunter JO, Reynolds PD and Tuffnell Q (1996) An open-label phase I/II dose escalation study of the treatment of pancreatic cancer using lithium gammalinolenate. *Anticancer Res* **16**: 867–874

Folch J, Lees M and Sloane-Stanley GA (1957) A simple method for the isolation and purification of total lipides from animal tissues. *J Biol Chem* **226**: 497–509

Hassam AG and Crawford MA (1978) The incorporation of orally administered radiolabeled dihomo γ-linolenic acid (20:3ω6) into rat tissue lipids and its conversion to arachidonic acid. *Lipids* **13**: 801–803

Honn KV, Tang DG, Gao X, Butovich IA, Liu B, Timar J and Hagmann W (1994) 12-lipoxygenases and 12(s)-HETE: role in cancer metastasis. *Cancer Metastasis Rev* **13**: 365–369

Horrobin, DF (1994) Unsaturated lipids: a new approach to the treatment of cancer. In *Effects of Fatty Acids and Lipids in Health and Disease*, Galli C, Simopoulos AP and Tremoli E (eds) pp. 77–80. World Rev. Nutr. Diet Karger: Basle

Houchens DP and Overjera A (1991) Experimental therapy in subcutaneous transplants. In: *The Nude Mouse in Oncology Research*. Boven E and Winograd B (eds), pp. 133–147. CRC Press: Boca Raton, FL

© Cancer Research Campaign 1997 

Jiang WG, Hiscox S, Hallet MB, Horrobin DF, Mansel RE and Puntis CA (1995) Regulation of the expression of E-cadherin on human cancer cells by γ-linolenic acid (GLA). *Br J Cancer* **71**: 5043–5048

Jiang WG, Hiscox S, Puntis CA, Hallet MB, Bryce RP, Horrobin DF and Mansel RE (1996) Gamma linolenic acid inhibits tyrosine phosphorylation of focal adhesion kinase and paxillin and tumour cell matrix interaction. *Int J Oncol* **8**: 583–587

Marra CA and de Alaniz MJT (1992) Incorporation and metabolic conversion of saturated and unsaturated fatty acids in SK-Hep 1 human hepatoma cells in culture. *Mol Cell Biochem* **117**: 107–118

Morrison WR and Smith LM (1964) Preparation of fatty acid methyl esters and dimethylacetals from lipids with boron fluoride-methanol. *J Lipid Res* **5**: 600–608

Morton RE, Hartz JW, Reitz RC, Moseley Waite B and Morris H (1979) The acyl-CoA desaturase of microsomes from rat liver and the Morris 7777 hepatoma. *Biochim Biophys Acta* **573**: 321–331

Morton RE, Waite M, Lynn King V and Morris HP (1982) Uptake and metabolism of free fatty acids by the Morris 7777 hepatoma and host rat liver. *Lipids* **17**: 529–537

Osborne CK, Hobbs K, Clark GM (1985) Effect of estrogens and antiestrogens on growth of human breast cancer cells in athymic nude mice. *Cancer Res* **45**: 584–590

Pritchard GA, Jones DL and Mansel RE (1989) Lipids in breast carcinogenesis. *Br J Surg* **76**: 1069–1073

Sakai T and Yamaguchi N (1984) Prostaglandin D2 inhibits the proliferation of human malignant tumor cells. *Prostaglandins* **27**: 17–26

Takeda S, Horrobin DF and Manku MS (1992) The effects of gamma-linolenic acid on human breast cancer cell killing, lipid peroxidation and the production of schiff-reactive materials. *Med Sci Res* **20**: 203–205

van Hoeven RP and Emmelot P (1973) Plasma membrane lipids of normal and neoplastic tissues. In: *Tumor Lipids: Biochemistry and Metabolism*, Wood R (ed.), pp. 126–138. AOCS Press: Champaign, IL

Voss AC and Sprecher H (1988) Metabolism of 6,9,12-octadecatrienoic acid and 6,9,12,15-octadecatetraenoic acid by rat hepatocytes. *Biochim Biophys Acta* **958**: 153–162

Ziboh VA (1996) The biological/nutritional significance of γ-linolenic acids in the epidermis: metabolism and generation of potent biological modulators. In *γ-linolenic Acid: Metabolism and its Roles in Nutrition and Medicine*, Huang YS and Mills DE (eds.), pp 118–136. AOCS Press: Champaign, IL

© Cancer Research Campaign 1997